# Development of a semi-open chamber system for the gas exchange measurement of whole-canopy under steady and unsteady states in cucumber seedlings

**DOI:** 10.1186/s13007-023-01059-1

**Published:** 2023-08-07

**Authors:** Yu Hyun Moon, Ui Jeong Woo, Ha Seon Sim, Tae Yeon Lee, Ha Rang Shin, Jung Su Jo, Sung Kyeom Kim

**Affiliations:** 1 Department of Horticultural Science, College of Agricultural and Science, Kyungpook National University, Daegu, 41566 Korea; 2 Institute of Agricultural Science and Technology, Kyungpook National University, Daegu, 41566 Korea

**Keywords:** CO_2_ assimilation rate, Cucumber seedlings, Gas exchange rate, Photosynthesis model, Semi-open chamber, Whole-canopy

## Abstract

**Background:**

Large-scale data on the photosynthetic characteristics of whole crop canopy is crucial for improving yield. However, current data collection methods remain challenging, and the time constraints associated with photosynthetic data collection further complicate matters. Developing a practical yet easy-to-use tool for collecting whole-canopy data is essential to address these challenges. Furthermore, it is necessary to obtain instantaneous measurements of photosynthetic rate over a wide range of CO_2_ concentrations under an unsteady state to enable faster data collection and obtain reliable biochemical limits of carbon assimilation. This study developed a semi-open chamber system with steady and unsteady state measurement techniques to collect biochemical photosynthetic data from an entire cucumber canopy, emphasizing the correction procedures for CO_2_ concentration of unsteady state measurements applicable regardless of chamber scale.

**Results:**

After constructing a semi-open chamber system, we described how to correct measurement errors according to chamber volume. In order to assess the accuracy of the newly developed system, an analysis was conducted to determine the overall measurement error resulting from variations in the reference, sample CO_2_ concentration, and leakage flow rate. The total measurement error was accurate to no more than 10%. Furthermore, the difference between the photosynthetic rate of the single leaf and that of the whole-canopy was not significant in Rubisco activity-limited carboxylation range. In addition, the Farquhar–von Caemmerer–Berry (FvCB) model parameters and the photosynthetic rate estimation values were compared to evaluate the steady- and unsteady state measurement methods between the cucumber seedlings' single-leaf and whole-canopy. The average root mean square error of the FvCB model in the steady (standard A-C_i_ response) and unsteady states (800 to 400 ramp) of the chambers was 1.4 and 2.3, respectively. Results show that the developed system is suitable for measuring the gas exchange rate of the cucumber canopy.

**Conclusions:**

We demonstrate the correction method for measurement errors to enable the gas exchange rate of the whole-canopy even in an unsteady state. The correction method of the measurement system of the gas exchange rate for the whole- canopy can be applied regardless of the volume of the chamber, and it can be applied simply to other chamber systems. In addition, an unsteady state measurement method for fast data collection was also applicable. However, it was deemed necessary to identify a more optimal measurement range by conducting measurements across a broader range of values.

**Supplementary Information:**

The online version contains supplementary material available at 10.1186/s13007-023-01059-1.

## Background

Large-scale screenings such as phenomics of plant phenotypes are required in food security and biofuel or cereal crop production [1]. Photosynthetic rate measurement can screen for desired traits in plants by assessing physiological functions or reactions [2]. The integrated CO_2_ assimilation rate of all leaves inside a canopy positively correlates to crop yields [3, 4]. Modeling via photosynthetic rate measurement is mainly used to assess the environmental feedback of plants and estimate the future vegetative carbon uptake [5]. Previous studies have estimated the photosynthetic rate of whole crop canopy using various modelings and techniques. The Farquhar–von Caemmerer and Berry (FvCB) biochemical model is mainly used at the leaf level because it can reflect various environmental factors [6].

Further, many models of the photosynthetic rate of whole crop canopy, which reflect the structural characteristics of plants and the scattering of light within the canopy, have been studied [7–11]. The measurement of physiological traits in real-time is commonly achieved through leaf-level gas exchanges [12]. However, to date, an easy-to-use and concise yet effective tool has not been developed for screening and measuring the photosynthetic rate of whole crop canopy [13].

Two main types of chamber systems are available for measuring the gas exchange rate: open chamber systems [14–19] and closed chamber systems [17, 20–22]. The latter has a simple structure. However, in the leaf chamber, CO_2_ concentration decreases, and humidity increases because of transpiration and photosynthesis without introducing ambient air, rendering it challenging to measure the gas exchange rate continuously and accurately under dynamic environmental conditions. A closed whole-canopy chamber system has been developed to measure the gas exchange rate constantly; however, such continuous measurement has its limits [13, 21]. Although an open chamber system can compensate for the shortcomings of a closed system, the system is complex [23].

Moreover, several problems may arise in the case of an open chamber system during the gas exchange rate measurement of the whole crop canopy. Depending on system performance, there may be a difference in accuracy and precision degree. When a chamber is more extensive, errors may still occur in the measured and calculated values of the photosynthetic rate owing to the residence time of the air in the chamber [19]. In addition, it may be more challenging to measure the gas exchange rate under dynamic environmental conditions and unsteady states. This error could be exacerbated because the structure of an open chamber system requires two infrared gas analyzers (IRGA). A more detailed classification (semi-open and semi-closed) of chamber types can be based on the following characteristics: a semi-type injects gas of the desired CO_2_ concentration into a chamber using a CO_2_ cylinder rather than in the atmosphere. A semi-type chamber system has the disadvantage of adding a device to regulate the CO_2_ concentration, increasing the system’s complexity.

Portable gas exchange systems (LI-6800, Li-Cor Co., Inc., Lincoln, NE, U.S.A.) have been used to measure the gas exchange rate at the leaf level under unstable environmental conditions [2, 5, 12]. Instantaneously measuring the gas exchange rate over a wide range of CO_2_ concentrations in an unsteady state could rapidly collect large-scale data on biochemical photosynthetic models, providing the biochemical limitations of carbon assimilation. Notably, the gas exchange rate can be measured in an environment that continuously changes the concentration of carbon dioxide around the entire crop canopy. The larger the chamber volume, the more dynamic and unstable the environmental conditions. Therefore, measuring the gas exchange rate of the whole crop canopy under a dynamic and unstable state is essential.

In this study, a new semi-open chamber system is developed to measure the gas exchange rate of the whole crop canopy. A correction method is devised that could be applied regardless of the chamber scale to use the steady and unsteady state measurement methods. Notably, the distinctive features of this study are as follows: (1) during development, an error correction method that could be applied regardless of the scale of a chamber was presented. To date, few semi-open chamber systems have been developed, considering the problems during chamber scaling. In addition, unsteady state measurement methods cannot be applied with the existing correction. (2) this is the only study to successfully apply the unsteady state measurement method of the whole crop canopy. The developed method has been evaluated by focusing on the FvCB model parameters.

## Materials and methods

### Components of a semi-open chamber system

**Fig. 1:**
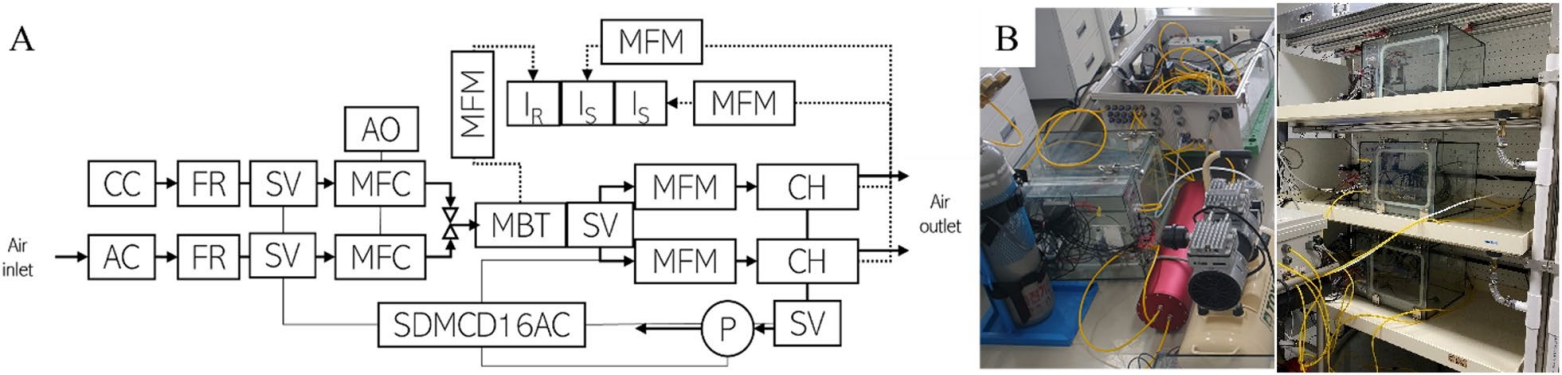
Schematic (**A**) (*AC* an air compressor, *CC* a CO_2_ cylinder, *SV* a solenoid valve, *AO* the analog output, *MBT* a mixed buffer tank, *MFM* a mass flow meter, *P*: a pump, *CH* a chamber, *I*_*.R*._an infrared gas analyzer of the reference CO_2_, *I*_*s*_ an infrared gas analyzer of the sample CO_2_) and images (**B**) of the semi-open chamber system for the gas exchange rate measurement of the whole crop canopy: The supplied air passed through a desiccant and soda lime. The desiccant was connected to a three-way valve, and a flow rate regulator could adjust the relative humidity of the air before passing the desiccant. CC was used to create the desired CO_2_ concentration in the CH. MFC controlled the flow rate of CO_2_-free air from AC and pure CO_2_ from CC. MBT was used for agitation of CO_2_-free air and pure CO_2_ air by air flow rate. MFM was used to check and control the airflow rate entering the chambers

A semi-open chamber system is a setup for continuous measurement of the gas exchange rate; it comprises a three-layered shelf inside a closed transplant production system with artificial lighting. Figure 1 shows a schematic of a semi-open chamber system. Two chambers of semi-open type (A and B) comprising 5-mm-thick clear glass with a 400 $\times$ 405 mm inner ground area and a height of 335 mm, giving a volume of 54 L, were used herein. The air inside the chamber was supplied with mixed air through the buffer tank (9 L) composed of the desired CO_2_ concentration and was continuously circulated using three small fans (0.5 m s^−1^) with a cooling coil to control the air temperature attached to the chamber side. When the air temperature was maintained at 25 °C, the air temperature of the chamber was kept at 27.3 $\pm$ 0.4 °C. Above 28.0 °C, the air temperature was lowered by an operating cooling coil. The individual cooling coil from each chamber was controlled with a switching power relay (SDM16AC/DC, Campbell Scientific Inc., U.S.A.). A 12-V and a 220-V power source were sued to power the three small fans and the three coils, respectively. The ambient inlet air was sucked using an air compressor. Before entering the buffer tank, the CO_2_ was removed using soda lime, and the desiccant controlled the humidity with a bypass through the flow speed regulator. The CO_2_ gas cylinder supplied the CO_2_ gas in the mixed air. CO_2_ concentration was regulated by controlling the flow rate with mass flow controllers (MC-10SLPM-D and MC-50SCCM, Alicat Scientific Inc., U.S.A.). It was then allowed to manipulate the set CO_2_ concentration.

Because a semi-open system enables a continuous flow rate, the chamber does not have to be airtight. The inlet mixed air flow rate was 10 L min^−1^ through mass flow controllers to each chamber. An air pump (N811KTDC, K.N.F. Neuberger Ltd., South Korea) was used to maintain the outlet air flow rate at 9 L min^−1^ outside each chamber. The gas of a reference infrared gas analyzer (IRGAr; Li-850, Li-Cor Co., Inc., Lincoln, NE, U.S.A.) was sucked from the buffer tank using a flow meter at 1 L min^−1^. Likewise, the gas of sample IRGAs (Li-840, Li-Cor Co., Inc., Lincoln, NE, U.S.A.) was sucked from each chamber using another flow meter. Each IRGA was calibrated with two standard gases (null gas, 600 μmol mol^−1^ CO_2_/N_2_ buffer) before setting up the system.

The air temperature and relative humidity were measured with a probe (HMP60, Vaisala Inc., Finland) and full spectrum with quantum sensors (SQ-500-SS, Apogee Instruments Inc., U.S.A.) inside each chamber. A barometer (PTB110, Vaisala Inc., Finland) was used to measure the air pressure in each chamber. In addition, thermocouples (LT-1M, Bio Instruments S.R.L., Moldova) were used to measure the leaf temperature. All sensors, reference, and sample CO_2_ and H_2_O concentrations were recorded using a data logger (CR1000, Campbell Scientific Inc., U.S.A.) by parsing the digital output with a power relay (SDM16AC/DC, Campbell Scientific Inc., U.S.A.), which was used to control the power of the coils, air pumps, and solenoid valves. The data were collected every second, and the average environmental parameters were recorded every minute, hour, and day.

### Correction process to obtain the photosynthetic rate of whole crop canopy

The apparent net CO_2_ assimilation rate (*A*, in μmol CO_2_ m^−2^ s^−1^), transpiration rate (*E*, in mol H_2_O m^−2^ s^−1^), and the air molar flow rate (*flow*, in μmol s^−1^) were computed using the following equations (LI-COR LI-6400 Manual, 1998):

1 $${\varvec{A}}=\frac{{{\varvec{f}}{\varvec{l}}{\varvec{o}}{\varvec{w}}}_{{\varvec{i}}}\times \left({{\varvec{C}}}_{{\varvec{r}}}-{{\varvec{C}}}_{{\varvec{s}}}\right)}{100\times {{\varvec{L}}}_{{\varvec{a}}}}-{{\varvec{C}}}_{{\varvec{s}}}\times {\varvec{E}}+\frac{{{\varvec{f}}{\varvec{l}}{\varvec{o}}{\varvec{w}}}_{{\varvec{a}}}\times \left({{\varvec{C}}}_{{\varvec{a}}}-{{\varvec{C}}}_{{\varvec{s}}}\right)}{100\times {{\varvec{L}}}_{{\varvec{a}}}}$$

2 $${\varvec{E}}=\frac{{{\varvec{f}}{\varvec{l}}{\varvec{o}}{\varvec{w}}}_{{\varvec{i}}}\times \left({{\varvec{W}}}_{{\varvec{s}}}-{{\varvec{W}}}_{{\varvec{r}}}\right)}{100\times {{\varvec{L}}}_{{\varvec{a}}}\times (1000-{{\varvec{W}}}_{{\varvec{s}}})}$$

3 $${\varvec{flow}} = \frac{{\user2{ air }\,\user2{flow }\,{\varvec{rate}} \times {\varvec{P}} \times 10^{6} }}{{{\varvec{n}} \times {\varvec{R}} \times \left( {{\varvec{t}} + 273.15} \right) \times 60}}$$

where *flow*_*i*_ and *flow*_*a*_ denote the molar flow rate (μmol s^−1^) of the inlet and leakage air converted from the air flow rate (L min^−1^), respectively; *C*_*r*_ and *W*_*r*_ denote the CO_2_ (μmol CO_2_ mol^−1^) and H_2_O (mmol H_2_O mol^−1^) concentrations before entering the chamber, respectively; *C*_*s*_ and *W*_*s*_ denote the CO_2_ (μmol CO_2_ mol^−1^) and H_2_O (mmol H_2_O mol^−1^) concentrations in the chamber, respectively; *C*_*a*_ represents the ambient CO_2_ (μmol CO_2_ mol^−1^) concentrations; *L*_*a*_ represents the total leaf area (cm^2^) of whole crop canopy; *P* denotes the air pressure (atm); $n$ denotes the total amount of ideal gas measured in moles; R denotes the universal gas constant (0.08206 atm L mol^−1^ K^−1^); and t denotes the air temperature (°C).

The measured values of $A$ were computed by the difference between *C*_*r*_ and *C*_*s*_. The following measurement errors [2] increase with increasing chamber volume: (1) the amount by which the *C*_*s*_ lag *C*_*r*_ owing to residence time in the chamber; (2) the offset between *C*_*r*_ and *C*_*s*_ that may accumulate as *C*_*r*_ increases (or decreases); and (3) the residual time error caused by length differences in the path of reaching each IRGA of Cr and Cs. The third error, which amounts to less than a few tenth micromoles of CO_2_ m^−2^ s^−1^ in the system, can be neglected, whereas the others must be corrected to measure the photosynthetic rate. The second error worsens with increasing chamber volume because the time taken to increase the CO_2_ concentration of the chamber is considerably less than the time taken to increase the CO_2_ concentration of the buffer tank.

The measurement errors of the system were evaluated under empty chamber conditions to correct the increase in CO_2_ concentration in the chamber (*Ramp*_*chamber*_, μmol CO_2_ mol^−1^ s^−1^). *Ramp*_*in*_ and *Ramp*_*out*_ denote the molar ramping rate (μmol CO_2_ mol^−1^ s^−1^) of the CO_2_ concentration of the inlet and outlet, respectively. The incoming CO_2_ concentration from the buffer tank to the chamber acts as the mixing volume, diluting the outcoming CO_2_ concentration and delaying the measurement of ${C}_{s}$. Therefore, a difference exists between *Ramp*_*in*_ and *Ramp*_*out*_, and measurement errors occur. *Ramp*_*in*_, *Ramp*_*out*_, and *Ramp*_*chamber*_ were computed using the following equations.

4 $${\varvec{Ramp}}_{{{\varvec{in}}}} = {\varvec{C}}_{{{\varvec{r}}\left( {\varvec{t}} \right)}} \times \frac{{{\varvec{flow}}_{{\varvec{i}}} \times 22.4}}{{10^{6} \times {\varvec{V}}_{{{\varvec{chmaber}}}} }}\user2{ }\left( {{\varvec{t}} \ge 1} \right)$$

5 $${\varvec{Ramp}}_{{{\varvec{out}}}} = {\varvec{C}}_{{\varvec{r}}} \user2{^{\prime}}_{{\left( {{\varvec{t}} - 1} \right)}} \times \frac{{{\varvec{flow}}_{{\varvec{i}}} \times 22.4}}{{10^{6} \times {\varvec{V}}_{{{\varvec{chmaber}}}} }}\user2{ }\left( {{\varvec{t}} \ge 1} \right)$$

6 $${\varvec{Ramp}}_{{{\varvec{chamber}}}} = {\varvec{Ramp}}_{{{\varvec{in}}}} - {\varvec{Ramp}}_{{{\varvec{out}}}}$$

Here, *C*_*r(t)*_ denotes the CO_2_ concentration (μmol CO_2_ mol^−1^) entering the chamber at measurement time (*t*); *C*_*r(0)*_ represents 0 μmol CO_2_ mol^−1^ assuming there is no leakage in the chamber; *C*_*r(t-1)*_ denotes the corrected CO_2_ concentration (μmol CO_2_ mol^−1^) in the chamber at measurement time (t-1); and *V*_*chamber*_ denotes the volume (L) of the chamber.

*C*_*r*_ can be used when the CO_2_ concentration of the chamber is stable, as it is measured immediately before entering the chamber. Still, because of the above error, CO_2_ concentration is not indicated before entering the chamber under an unsteady state. Therefore, *C*_*r*_*'* is the corrected *C*_*r*_ (μmol CO_2_ m^−2^ s^−1^) indicated before the CO_2_ concentration enters the chamber. The leakage molar flow rate (*flow*_*a*_, in μmol s^−1^) is needed to quantify the chamber leakages to calculate $A$. Thus, *A'* is the corrected *A* (μmol CO_2_ m^−2^ s^−1^), which is a composite of net assimilation by the crop and the system kinetics and offsets described above. *C*_*r*_*'*, *flow*_*a*_, and *A'* were computed using the following equations:

7 $${\varvec{C}}_{{\varvec{r}}} \user2{^{\prime}}_{{\varvec{t}}} = {\varvec{C}}_{{\varvec{r}}} \user2{^{\prime}}_{{\left( {{\varvec{t}} - 1} \right)}} + {\varvec{Ramp}}_{{{\varvec{chamber}}}} \user2{ }\left( {{\varvec{t}} \ge 1} \right)$$

8 $${\varvec{flow}}_{{\varvec{a}}} = \frac{{22.4 \times \left( {{\varvec{flow}}_{{{\varvec{out}}}} \times {\varvec{C}}_{{\varvec{s}}} - {\varvec{flow}}_{{{\varvec{in}}}} \times {\varvec{C}}_{{\varvec{r}}} } \right)}}{{10^{6} \times {\varvec{V}}_{{{\varvec{chmaber}}}} \user2{ } \times ({\varvec{C}}_{{\varvec{a}}} - {\varvec{C}}_{{\varvec{r}}} \user2{^{\prime}})}}$$

9 $$\user2{A^{\prime}} = \frac{{{\varvec{flow}}_{{\varvec{i}}} \times \left( {{\varvec{C}}_{{\varvec{r}}} {\mathbf{^{\prime}}} - {\varvec{C}}_{{\varvec{s}}} } \right)}}{{100 \times {\varvec{L}}_{{\varvec{a}}} }} - {\varvec{C}}_{{\varvec{s}}} \times {\varvec{E}} + \frac{{{\varvec{flow}}_{{\varvec{a}}} \times \left( {{\varvec{C}}_{{\varvec{a}}} - {\varvec{C}}_{{\varvec{s}}} } \right)}}{{100 \times {\varvec{L}}_{{\varvec{a}}} }},$$

where *flow*_*out*_ denotes the molar flow rate (μmol s^−1^) of outlet air converted from the air flow rate (L min^−1^).

The correction procedures explained earlier required the following several conditions [2]: (1) all conditions (flow rate, temperature, ramp range, direction, and ramp rate) must be the same for measurement. (2) The exact match IRGA should be used for the empty chamber and plant curves. (3) Under original conditions, the $A$ value of the empty chamber should be close to zero, and the sample and reference CO_2_/H_2_O mole fractions should be approximately equal. (4) Which best fits the empty chamber correction curve and what range of CO_2_ concentration is useable must be determined. (5) The differential water mole fraction between the correction and measurement runs must be minimized. (6) The appropriate range of CO_2_ concentration and CO_2_ ramping rate for the parameters of interest must be determined, and preliminary tests on various environmental conditions of interest must be run. Therefore, the correction was performed in a closed transplant production system capable of artificial environment control to satisfy these criteria.

### Precision check through total error estimation

The total measurement error (*e*_*t*_) of the developed system was evaluated under the steady state of the chambers. A partial derivative was used to estimate et caused by changes in reference, sample CO2 concentration, and leakage rate to verify precision. *e*_*t*_ was computed using the following equations.

10 $${\varvec{e}}_{{\varvec{t}}} = \user2{A^{\prime}}\left( {{\varvec{k}},{\varvec{C}}_{{\varvec{r}}}^{\user2{^{\prime}}} ,{\varvec{C}}_{{\varvec{s}}} } \right) - \user2{A^{\prime}}\left( {{\varvec{k}}_{0} ,{\varvec{C}}_{{{\varvec{r}}\left( 0 \right)}}^{\user2{^{\prime}}} ,{\varvec{C}}_{{{\varvec{s}}\left( 0 \right)}} } \right)$$

11 $$\user2{A^{\prime}}\left( {{\varvec{k}},{\varvec{C}}_{{\varvec{r}}}^{\user2{^{\prime}}} ,{\varvec{C}}_{{\varvec{s}}} } \right) - \user2{A^{\prime}}\left( {{\varvec{k}}_{0} ,{\varvec{C}}_{{{\varvec{r}}\left( 0 \right)}}^{\user2{^{\prime}}} ,{\varvec{C}}_{{{\varvec{s}}\left( 0 \right)}} } \right) = {\varvec{A}}\left( {{\varvec{k}}_{0} + \Delta {\varvec{k}},{\varvec{C}}_{{{\varvec{r}}\left( 0 \right)}}^{\user2{^{\prime}}} + \Delta {\varvec{C}}_{{\varvec{r}}}^{\user2{^{\prime}}} ,{\varvec{C}}_{{{\varvec{s}}\left( 0 \right)}} + \Delta {\varvec{C}}_{{{\varvec{s}}\left( 0 \right)}} } \right) - {\varvec{A}}\left( {{\varvec{k}}_{0} ,{\varvec{C}}_{{{\varvec{r}}\left( 0 \right)}}^{\user2{^{\prime}}} ,{\varvec{C}}_{{{\varvec{s}}\left( 0 \right)}} } \right)$$

12 $${\varvec{e}}_{{\varvec{t}}} \user2{ } \approx \frac{{\partial {\varvec{A}}}}{{\partial {\varvec{k}}}} \cdot \Delta {\varvec{k}} + \frac{{\partial {\varvec{A}}}}{{\partial {\varvec{C}}_{{\varvec{r}}} \user2{^{\prime}}}} \cdot \Delta {\varvec{C}}_{{\varvec{r}}} \user2{^{\prime}} + \frac{{\partial {\varvec{A}}}}{{\partial {\varvec{C}}_{{\varvec{s}}} }} \cdot \Delta {\varvec{C}}_{{\varvec{s}}}$$

### Evaluation of the photosynthetic rate of whole-canopy in the steady and unsteady state

This study used cucumber (‘Joenbaekdadagi') seedlings planted in six 4 × 5 cell trays 10 days after emergence and cultivated in a closed transplant production system. During *A* measurements, the air temperature and relative humidity inside the closed transplant production system were maintained at 23°C and 60%, respectively. The chamber air temperature and relative humidity were maintained at 25 °C and 70%, respectively. The light intensity in the chambers was maintained at 400 μmol m^−2^ s^−1^. After the correction of the gas exchange measurement system, gas exchange rate measurements were performed in two states: (1) steady and (2) unsteady states of CO_2_ concentrations in the chamber.

The order of measurements is known to influence the results, as evidenced by the common practice of carefully returning to a standard, an intermediate value between the low and high CO_2_ ranges of standard A-C_i_. Therefore, under a steady state, photosynthetic rate measurements were maintained stepwise through eleven levels, i.e., 0, 100, 200, 300, 400, 500, 600, 700, 800, 900, and 1000 μmol CO_2_ mol^−1^. Under an unsteady state, the CO_2_ ramping rates ran in two CO2 directions, increasing and decreasing. [24] recommended limiting the CO_2_ ramping rates to 100 μmol CO_2_ mol^−1^ min^−1^. Owing to the large volume of the chambers, the CO_2_ ramping rate was limited in the range of around 15–33 μmol CO_2_ mol ^−1^ min^−1^. Herein, the CO_2_ concentration was changed at a limiting CO_2_ ramping rate starting at 0 μmol CO_2_ mol^−1^ and from 0 to 500, 800, and 1000 μmol CO_2_ mol^−1^ s^−1^ 30 min^−1^. Moreover, the CO_2_ concentration was changed from 800 to 400 μmol CO_2_ mol^−1^ s^−1^ 30 min^−1^ (looped ramp measurement under a steady state at 800 μmol CO_2_ mol^−1^). All measurements began at 0 μmol CO_2_ mol^−1^ (*C*_*r(0)*_) to compute *C*_*r*_ in the chamber. In addition, a portable photosynthesis system (LI6400XT, Li-Cor Co., Inc., Lincoln, NE, U.S.A.) was used to compare the photosynthetic rate at leaf and whole-canopy. The photosynthetic rate of cucumber seedling leaves was measured 11 days after emergence under the same environmental conditions as the entire canopy.

The measurement results were compared by applying the FvCB model that reflects the biochemical characteristics of cucumber seedlings. The parameters used for comparison were Vc_max_ and J_max_, which denote the maximum rate of Rubisco activity-limited carboxylation and the maximum value of the electron transport rate under saturated light, respectively.

### Data analysis

Statistical analysis was conducted with analyses of variance using the S.A.S. program (SAS 9.4, S.A.S. Institute Inc., NC, U.S.A.) was used to conduct statistical analysis with ANOVA among *A-C*_*i*_ responses under the steady and unsteady states. Tuckey’s honestly significant difference (HSD) test was used to examine significant differences (p-value = 0.05). According to the measurement methods used herein, the FvCB model was compared through root mean square error (RMSE) (Eqs. 13). All data on CO_2_ response were collected from cucumber seedlings planted in 4 × 5 cell trays of three biological replicates.

13 $${\varvec{RMSE}} = \user2{ }\sqrt {\frac{1}{{\varvec{n}}}\mathop \sum \limits_{{{\varvec{i}} = 1}}^{{\varvec{n}}} \left( {{\varvec{y}}_{{\varvec{i}}} - \hat{\user2{y}}_{{\varvec{i}}} } \right)^{2} }$$

where ${{\varvec{y}}}_{{\varvec{i}}}$ denotes the predicted ith apparent net CO_2_ assimilation rate of the FvCB model; ${\widehat{{\varvec{y}}}}_{{\varvec{i}}}$ denotes the measured ith apparent net CO_2_ assimilation rate of the system; n denotes the number of pairs between the predicted and measured result.

## Results

### Precision evaluation of the development system

**Fig. 2:**
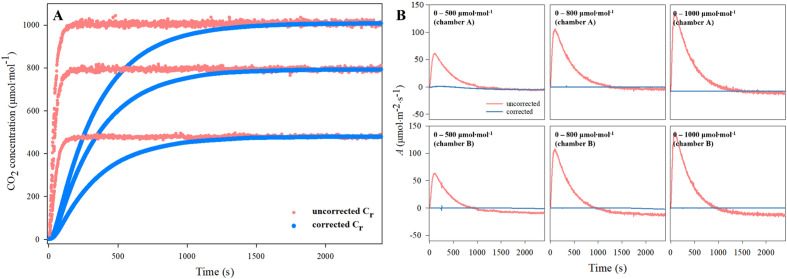
**A** Uncorrected reference CO_2_ concentration (*C*_*r*_) and corrected reference CO_2_ concentration (*C*_*r*_*'*) according to a desired CO_2_ concentration (500, 800, and 1000 mmol CO_2_ mol^−1^) of the empty chamber **A** and **B** Uncorrected and corrected net CO_2_ assimilation rate of empty chambers A and B at each uncorrected (*C*_*r*_) and corrected reference CO_2_ concentration (*C*_*r*_*'*)

*C*_*r*_ of the empty chamber was measured at both steady and unsteady states. Subsequently, the data obtained from both states were subjected to a correction process. All *C*_*r*_ before correction overestimated the CO_2_ concentration in the chambers (Fig. 2A) because of the errors raised earlier. A slight difference in values between *C*_*r*_ and* C*_*s*_ was observed in both the steady and unsteady states. The dilution in CO_2_ concentration determined this difference by leakage and volume of the chambers. Post correction, *C*_*r*_*'* could match the CO_2_ concentration in both chambers to the target concentration. When the target CO_2_ concentration was 500, 800, and 1000, the mean *C*_*r*_*ʹ* was 999.8, 800.0, and 500.1 μmol CO_2_ mol^−1^, respectively. Each has a standard deviation of 0.2, 0.1, and 1.5 μmol CO_2_ mol^−1^. All the *C*_*r*_*'* showed a nonlinear increase regardless of the CO_2_ concentration.

When the CO_2_ concentration of the chambers was changed, a significant difference in *A* was confirmed owing to the measurement error of *C*_*r*_ (Fig. 2B). At CO_2_ concentrations of 500, 800, and 1000 μmol CO_2_ m^−2^ s^−1^ of the chambers, it appeared at maximum as 63.2, 106.1, and 134.2 μmol CO_2_ m^−2^ s^−1^ difference of the chamber A, respectively. In the case of chamber B, each difference showed as much as a maximum of 63.2, 108.2, and 136.4 μmol CO_2_ m^−2^ s^−1^: the more significant the CO_2_ concentration changes, the larger the difference. After correction, the *A'* of chamber A in each CO_2_ concentration was < 0.1 μmol CO_2_ m^−2^ s^−1^, whereas that of chamber B was < 0.5 μmol CO_2_ m ^−2^ s^−1^.

**Fig. 3:**
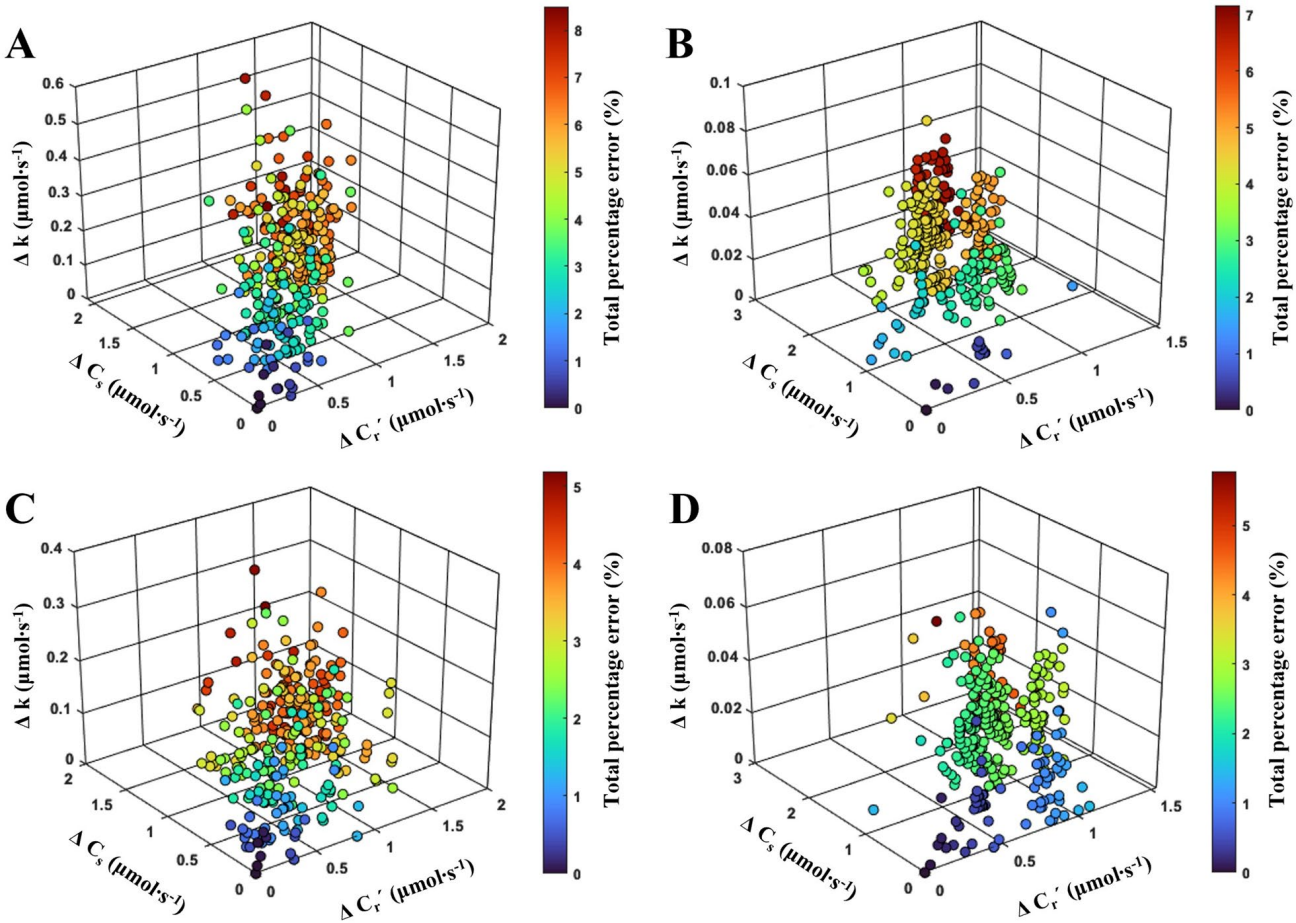
**A**, **C** Total measurement error (*e*_*t*_) of *A* of cucumber seedlings at 10 days after sowing in chambers **A** and **B** at 500 mmol CO_2_ mol^−1^ and (**B**, **D**) 1000 mmol CO mol^−1^ for 300 s

As shown in Fig. 3, three factors (*C*_*r*_*'*, *C*_*s*_, and *k*) could cause the *e*_*t*_ of *A'* measured at the steady state. The mean and standard deviations were 4.15% and 1.9%, respectively, at 500 μmol CO_2_ mol^−1^ in chamber A. The mean and standard deviations were 4.15% and 1.5%, respectively, in the case of 1000 μmol CO_2_ mol^−1^. The maximum *e*_*t*_ were 8 and 7.2%, respectively. In chamber B at 500 and 1000 μmol CO_2_ mol^−1^, the mean *e*_*t*_ was 2.8% and 2.4%, and maximum *e*_*t*_ was only 5.2% and 5.8%, with standard deviations of 1.2% and 1.1%, respectively. Even though chamber B had a higher leakage than chamber A, it showed a relatively more minor *e*_*t*_, and *e*_*t*_ of both chambers did not exceed 10%. Given the cucumber seedlings' rapid response to microenvironmental changes that could affect the measured values' accuracy, it was concluded that the semi-open chamber system provided sufficient precision for measuring the photosynthetic rate of the entire cucumber seedlings.

### Comparison of photosynthesis rate at leaf and whole-canopy

The measurement values of photosynthetic rate in the steady state were compared between leaf and whole-canopy (Table 1). At reference CO_2_ concentrations of 200 and 400 μmol CO_2_ mol^−1^, there was no significant difference in leaf and whole-canopy photosynthetic rate. Differences in photosynthetic rates between the leaf and whole-canopy systems began to appear at reference CO_2_ concentrations above 500 μmol CO_2_ mol^−1^ (not shown in Table 1). The whole-canopy was formed on the tray with limited planting density. Still, because the seedlings were relatively young, the shading effect did not significantly reduce light interception, so it was thought that there was no significant difference in photosynthetic rates between the two. However, it was determined that further analysis of light interception was necessary to enhance the precision of the analysis.

**Table 1:** Net photosynthesis rate (*A*, mmol m^−2^ s^−1^) measured at 10 (whole-canopy chambers A and B) and 11 (portable photosynthesis system) days after the emergence of cucumber seedlings (n = 3) using steady state methods (standard A-C_i_ response)

Reference CO_2_ System type	0 (mmol CO_2_ mol^−1^)	200 (mmol CO_2_ mol^−1^)	400 (mmol CO_2_ mol^−1^)	600 (mmol CO_2_ mol^−1^)	800 (mmol CO_2_ mol^−1^)	1000 (mmol CO_2_ mol^−1^)
Portable leaf chamber system	− 2.8c^z^	2.8a	7.3a	10.0a	11.5a	12.7a
Whole-canopy chamber A	− 2.4b	3.1a	7.6a	9.4b	10.8b	11.8b
Whole-canopy chamber B	− 2.1a	3.3a	7.2a	9.1b	10.7b	11.9b

^z^ Identical lower case letter indicates that means are not significantly different according to Tuckey’s HSD test (p > 0.05). Different lower case letters indicate that means are significantly different according to Tuckey's HSD test (p > 0.05)

### Measurement of photosynthetic rate for steady and unsteady state

**Fig. 4:**
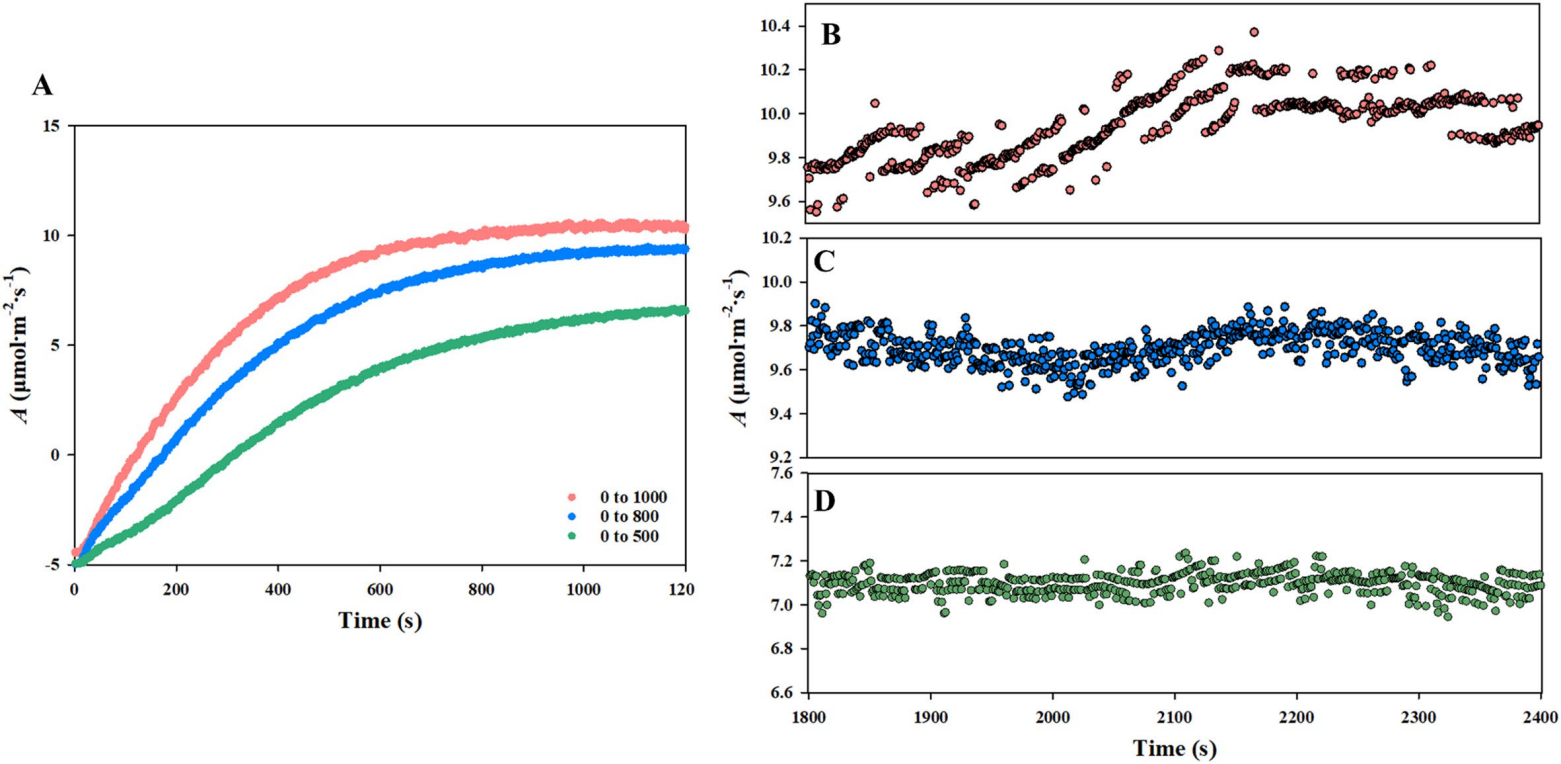
**A** Continuous measurement of *A′* of cucumber seedlings in chamber **A**, **B** measurement of *A* at 1000 mmol CO_2_ mol^−1^, **C** measurement of *A* at 800 mmol CO_2_ mol^−1^, and **D** measurement of *A* at the steady state at 500 mmol CO_2_ mol^−1^

The *A′* of the cucumber seedlings was measured at the unsteady state (ramping from 0 to 500, 800, and 1000 μmol CO_2_ mol ^−1^ s^−1^ 30 min^−1^). In the case of the unsteady state, the measured *A′* differed depending on the slope of the CO_2_ ramp (Fig. 4). As the CO_2_ ramp became more extensive, the measured *A'* significantly increased. In addition, the *C*_*i*_ (intercellular CO_2_ concentration, μmol CO_2_ mol^−1^) was calculated according to the method shown in [25]. However, considering the *C*_*i*_, unlike *A′* that appeared with time series, it was found that *A′ *that appeared with *C*_*i*_ series increased as the CO_2_ ramp was smaller. In addition, it was shown that *A* with *C*_*i*_ values increased as the slope of the CO_2_ ramp became lower. It was related to the ramp slope the whole-canopy could respond to by concentration dilution by the volume of the chamber. In the case of the steady state, the mean *A*′ of chamber A was 8.3, 10.8, and 11.8 μmol CO_2_ m^−2^ s^−1^ at the CO_2_ concentration 500, 800, and 1000 μmol CO_2_ mol^−1^ of *C*_*r*_*′*, respectively, whereas the mean *A*′ of the chamber B was 8.6, 10.7, and 11.9 μmol CO_2_ m^−2^ s^−1^. There was no significant difference from the values taken instantaneously with the unsteady state method at the same *C*_*r*_′.

### Comparison of the FvCB model for steady and unsteady state

**Fig. 5:**
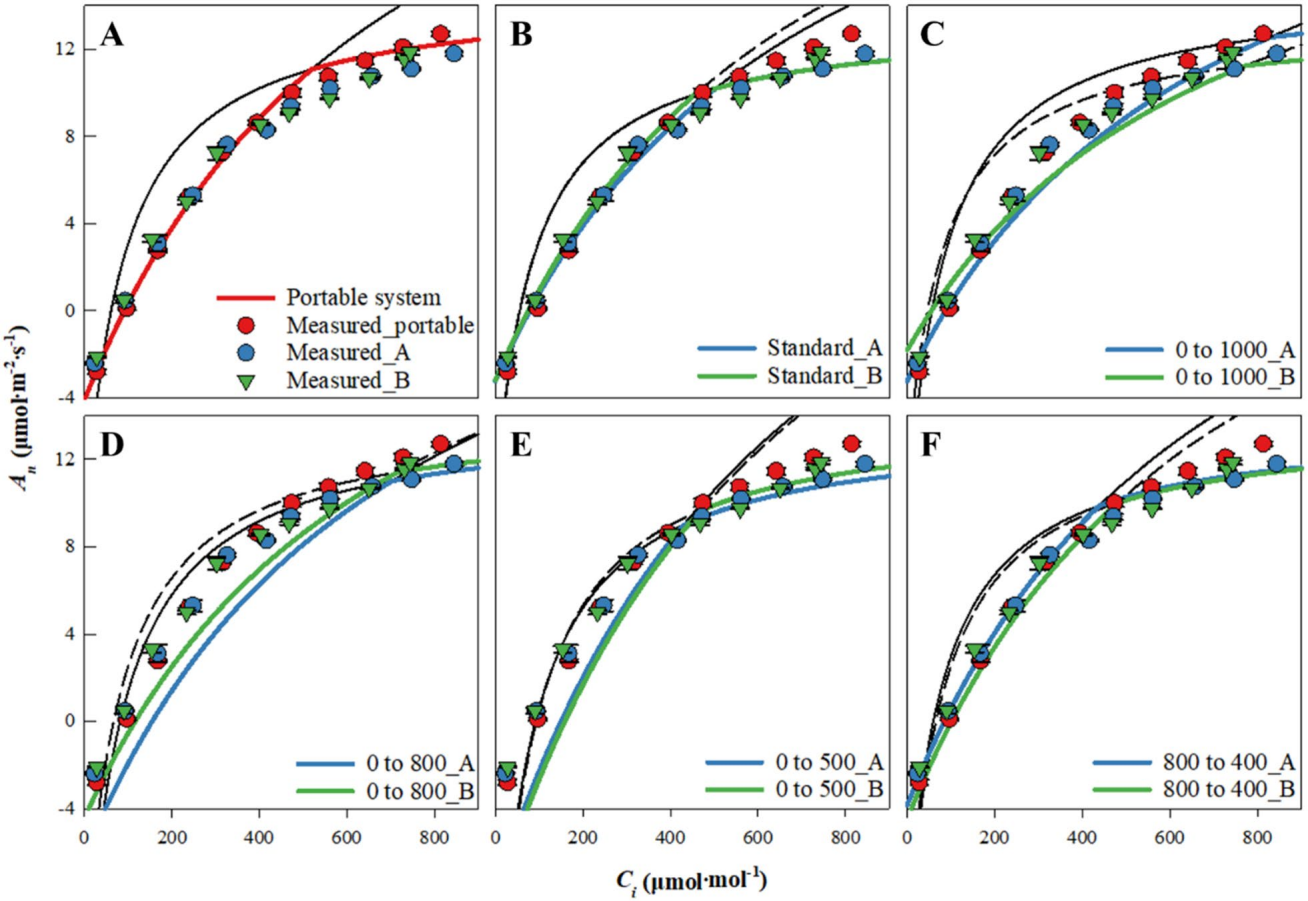
Comparison of the measured and estimated *A* (FvCB model) using steady and unsteady state methods in portable photosynthesis system, chamber **A** and **B** for cucumber seedlings’ A-C_i_ response (**A**, standard **A**-**C**_i_; **B**, 0–500 ramp; **C**, 0–800 ramp; **D**, 0–1000 ramp; **E**, 800–400 ramp). The FvCB model estimates *A*_*n*_ (net CO2 assimilation rate supported by Rubisco and RuBP-regeneration) for the **A**-**C**_i_ response by subtracting respiration rate from the smaller of A_c_ (CO_2_ assimilation rate supported by Rubisco) and A_j_ (CO_2_ assimilation rate supported by RuBP-regeneration). The black lines are values that were not adopted as the *A*_*n*_

The parameters and performance of the FvCB model were compared between the steady and unsteady state of photosynthetic rate measurement (Table 2) to adopt a measurement method for efficiently collecting data for the biochemical photosynthetic model. Figure 5 shows the *A*-*C*_*i*_ responses estimated through the FvCB model according to each measurement method based on the standard *A*-*C*_*i*_ values. The RMSE was the lowest in the steady state measurement for chamber A. Moreover, the 800–400 ramping rate (30 min) showed a lower RMSE than the others. The RMSE of the 0–800 ramping rate differed from other measurement methods. In the case of chamber B, the 800–400 ramping rate (30 min) showed a similar value of RMSE in the steady state measurement. Compared to the portable photosynthesis system, the model (800–400 ramping rate) performed worse, although not significantly. The measurement method of 0–1000, 0–800, and 0–500 ramping rates (30 min) was considered inefficient in both cases.

**Table 2:** Parameters and RMSE of the FvCB model for the A-Ci response at 10 days after the emergence of cucumber seedlings were measured using steady and unsteady state methods in a portable photosynthesis system, chambers A and B

Measurement type		Vc_max_^x^		J_max_		RMSE^y^	
Portable photosynthesis system		33.9	b^z^	99.6	d	0.3	e
Chamber A	Standard	30.6	cd	83.5	g	1.1	de
	0 to 1000	27.6	e	97.2	de	2.7	bc
	0 to 800	32.7	bc	114.9	c	6.9	a
	0 to 500	41.9	a	122.5	b	7	a
	800 to 400	33.9	b	89	f	1.9	cd
Chamber B	Standard	31.7	bcd	82.5	g	1.6	cde
	0 to 1000	23.6	f	74.5	h	3.9	b
	0 to 800	29.8	de	100.3	d	4.2	b
	0 to 500	42.5	a	134.1	a	7.4	a
	800 to 400	33.9	b	94.6	e	2.7	bc

^z^ Identical lowercase letters indicate that means are not significantly different according to Tuckey’s HSD test (p > 0.05). Different lower case letters indicate that means are significantly different according to Tuckey's HSD test (p > 0.05)

^y^ RMSE for each model was calculated through the values measured by the standard *A-Ci* response and the values estimated by the FvCB model

^x^ *Vc*_*max*_ maximum rate of Rubisco activity-limited carboxylation, *J*_*max*_ the maximum value of the rate of electron transport under saturated light

For Vc_max_ estimation, the methods that were not significantly different from the portable photosynthesis system were the 0–800 and 800–400 ramping rate methods for chamber A and the standard and 800–400 ramping rate methods for chamber B. For Jmax, there was a significant difference between the portable photosynthesis and development chamber systems. It was due to differences in photosynthetic rate between the value of measurement from leaf and whole-canopy in the RuBp regeneration range.

## Discussion

The *flow*_*a*_ of the development photosynthetic rate measurement system was quantified in the conventional method [25]. Its values were < 5 mmol mol^−1^. In addition, the portable photosynthesis system showed that the *flow*_*a*_ decreased as the *flow*_*i*_ increased, and the *flow*_*i*_ set in this experiment was sufficient to reduce the *flow*_*a*_. As the *flow*_*a*_ in the chambers increased, it was difficult to control the CO_2_ concentration; therefore, measuring the photosynthetic rate could be challenging. Quantifying *flow*_*a*_ and reflecting it in the correction process is essential to measure the accurate photosynthetic rate. If the photosynthetic rate is measured without quantifying the *flow*_*a*_ when using the development photosynthetic rate measurement system, the CO_2_ concentration in the chambers could not be accurately predicted, which may cause measurement errors. Therefore, this process is considered essential when developing a gas exchange rate measurement system.

The correction of the *C*_*r*_ is also essential. Owing to measurement errors, the *C*_*r*_ does not accurately represent the CO_2_ concentration in the chambers, even in the steady state. In addition, measurement errors would occur more seriously in this experiment’s development photosynthetic rate measurement system than in the Li-6800 (LI-COR Bioscience, U.S.A.). To correct this, the *C*_*r*_ was corrected, and it was confirmed that the corrected *C*_*r*_ matched the CO_2_ concentration of the chamber. In a previous study, after quantifying the delay time between the sample and reference CO_2_ concentration, the offset between the photosynthetic rate before and after the correction was obtained in an empty chamber, and measurement was performed in an unsteady state [2]. For the Li-6800, the delay can be quantified because the reference and sample CO_2_ concentration shows a linear increase. However, in the case of the development system, as the volume of the chambers is large, quantifying the measurement delay time could not be applied because it showed a nonlinear increase in the sample and reference CO_2_ concentration. Therefore, the correction process was through the calculation procedures shown in this experiment. The prerequisite was to proceed with the gas exchange rate measurement with zero reference CO_2_ concentration. Consequently, the concentration in the chamber was accurately predicted, and the corrected photosynthetic rate showed a numerical value close to zero (within a few tenths), as mentioned in a previous study [24].

**Fig. 6:**
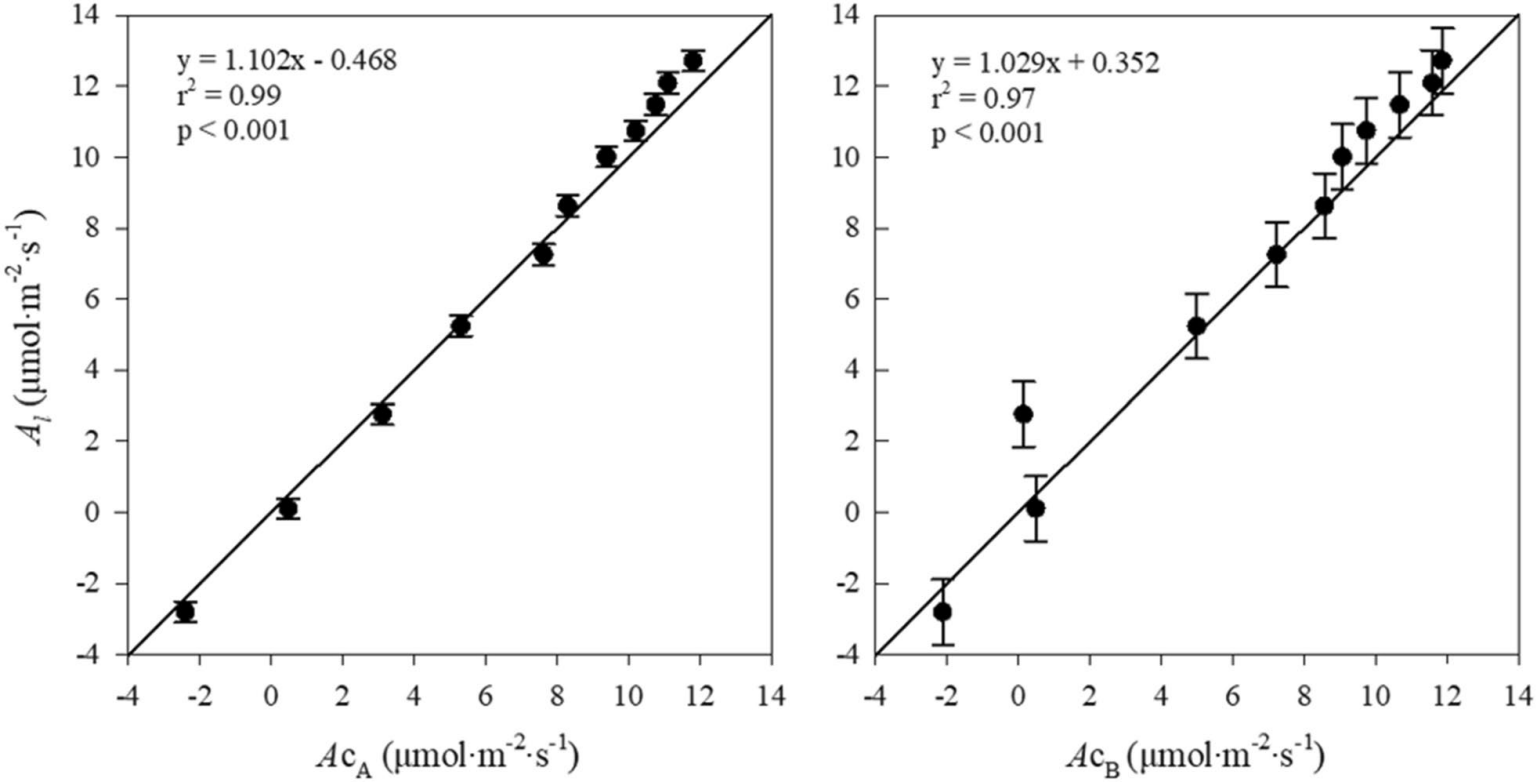
Relationship between the single leaf (A_l_) and whole-canopy (chamber **A**, *A*c_A_; chamber **B**, *A*c_B_) of the photosynthetic rate of cucumber seedlings according to the reference CO_2_ concentration in the chambers

The difference in the photosynthetic rate between cucumber seedlings’ single-leaf and whole-canopy was not significantly different (Fig. 6). There was no significant difference in the photosynthetic rate between the single-leaf and whole-canopy of papaya (‘Gran Golden’), with a coefficient of determination (r^2^) value of 0.95 observed for the one-to-one relationship [26]. A Plant with an open canopy architecture, such as papaya, could have similar values. Compared to single-leaf measured by the portable photosynthesis system, Chamber A showed a close linear relationship with an r2 value of 0.99, and Chamber B showed a value of 0.97. However, extending leaf-level measurements of photosynthetic rate to the whole-canopy level can vary depending on light interception due to leaf position and distribution [27, 28]. [29] showed that the photosynthetic rate of whole-canopy in cucumber seedlings decreased with decreasing light interception due to the shading effect of leaf morphology. This experiment determined that there were no shading effects due to the growth of cucumber seedlings. Moreover, it was concluded that additional light interception analysis based on the leaf area index would be necessary to improve the accuracy of the results.

The conventional photosynthesis measurement method proceeds with the measurement in a state where the chamber's environment is stabilized. Recently, [2] used the rapid *A*-*C*_*i*_ response method to show how to measure the *A*-*C*_*i*_ responses in 5 min. The advantage of this measurement method is that large-scale phenotyping for photosynthetic gas exchange parameters is possible within a short period, so it could be used to improve the photosynthetic model performance. Therefore, the same technique was applied to the development photosynthetic measurement system in this experiment and compared with the conventional measurement method. When comparing *A'* of the unsteady state and steady state measurements, unlike that of LI-6800 [2, 5], the significant difference was due to the CO_2_ ramp slope. In the case of LI-6800, it is deemed that the measured photosynthetic rate between the unsteady and steady state showed similar values even though it was more significant than the CO_2_ ramp slope used in this experiment because the chamber volume of LI-6800 was smaller. In addition, the leaf directly receives CO_2_ gas flow. Other than that, it may have been due to different crop varieties; however, it was not considered to have had a significant impact.

The comparison of A-C_i_ curves showed a relative difference between the measured values in this experiment, but their increasing and decreasing tendencies were similar. The *A′* in the unsteady state showed no significant difference from the *A′* in the steady state toward the end of the measurement time point. The CO_2_ ramp became smaller toward the endpoint due to the nonlinear increase of *C*_*r*_*′*. The leaves of the crop's whole-canopy could be given more time to respond because the CO_2_ concentration in the chamber does not change abruptly. Accordingly, it was confirmed that the *A′ *of the 800–400 ramping rate (30 min) showed similar values to that of the steady state measurement method, unlike other CO_2_ ramping rate methods. As in the case of the 800–400 ramping rate (30 min), if the appropriate CO_2_ ramping rate could be found, as shown in the rapid *A*-*C*_*i*_ response technique guide [24], it was judged that the standard A-Ci response in the measurement of the photosynthetic rate of the whole cucumber seedling canopy could measure the effective A-Ci responses.

The measurement of the 800–400 ramping rate (30 min) showed biochemical characteristics through the FvCB model, similar to the standard *A*-*C*_*i*_ responses in the portable photosynthesis system. In particular, in the lower CO_2_ ramping (13 μmol CO_2_ mol^−1^ min^−1^) slope for the unsteady state measurement, there was a similarity to standard *A*-*C*_*i*_ responses. In addition, widening the measurement range by varying the CO_2_ range within 15 μmol CO_2_ mol^−1^ min^−1^ of the ramping rate was necessary. The unsteady state measurement used in this experiment took about 30 min to collect data for *A*-*C*_*i*_ responses, but the standard method took 20 min per point, above 200 min. Thus, the unsteady state method measurement showed effective results for data collection required for the biochemical photosynthetic model, which could contribute to faster data collection. In addition, a semi-open chamber system could easily control the environment than a closed-chamber system [13, 23, 30]). Thus, if the correction process proceeds, it was determined to help continuously measure the gas exchange rates during the entire cultivation period of the whole crop canopy in dynamic environmental conditions.

## Conclusion

This study evaluated reference CO_2_ concentration, sample CO_2_ concentration, total measurement error, and photosynthetic rate. The photosynthetic model was also evaluated by comparing it to the measured values using the portable and developed photosynthesis systems. Until recently, almost all photosynthetic models have been used to estimate the crop's whole-canopy by measuring the photosynthetic rate of a leaf. It is less accurate than a direct measurement of the whole-canopy of the crop. In this study, the correction method of the measurement system of the gas exchange rate for the whole- canopy can be applied regardless of the volume of the chamber, and it can be applied simply to other chamber systems. In addition, an unsteady state measurement method for fast data collection was also applicable. However, it was deemed that finding a more efficient measurement range through measurement in a more extensive range is necessary.

### Supplementary Information

**Additional file 1:** The example of raw data for calculated the net photosynthetic rate using various parameters under 1000 μmol·mol^-1^ CO_2_ concentration inside chamber air.

## Data Availability

E original contributions presented in the study were included in the article/Additional file 1. Further inquiries can be directed to the corresponding author.
